# Irinotecan plus cisplatin followed by octreotide long-acting release maintenance treatment in advanced gastroenteropancreatic neuroendocrine carcinoma: IPO-NEC study

**DOI:** 10.18632/oncotarget.12900

**Published:** 2016-10-25

**Authors:** Jie Li, Ming Lu, Zhihao Lu, Zhongwu Li, Yiqiang Liu, Li Yang, Jian Li, Xiaotian Zhang, Jun Zhou, Xicheng Wang, Jifang Gong, Jing Gao, Yan Li, Lin Shen

**Affiliations:** ^1^ Department of GI Oncology, Key laboratory of Carcinogenesis and Translational Research (Ministry of Education), Peking University School of Oncology, Beijing Cancer Hospital & Institute, Beijing, China; ^2^ Department of Pathology, Key laboratory of Carcinogenesis and Translational Research (Ministry of Education), Peking University School of Oncology, Beijing Cancer Hospital & Institute, Beijing, China; ^3^ Center of clinical oncology, The University of Hong Kong-Shenzhen Hospital, Shenzhen, China

**Keywords:** gastroenteropancreatic neuroendocrine carcinomas, irinotecan, cisplatin, octreotide long-acting release, heterogeneous

## Abstract

There have been very few prospective studies of first-line chemotherapy on advanced gastroenteropancreatic neuroendocrine carcinoma (GEP-NEC). This phase II study assessed the activity and safety of irinotecan plus cisplatin (IP) followed by octreotide long-acting release (LAR) maintenance treatment in advanced GEP-NEC. Forty patients were treated and eighteen patients (45.0%) had a partial response. The median progression-free survival (PFS) and overall survival (OS) were 5.7 months and 12.9 months, respectively. Because GEP-NECs are heterogeneous, a subgroup analysis was conducted by dividing all patients into a high proliferation neuroendocrine tumor (NET) group (well differentiated neuroendocrine neoplasms with a Ki-67 level between 20-60%) or a poorly differentiated NEC (PDNEC) group. Compared with the PDNEC group, the patients in high proliferation NET group had a lower response rate (0% *versus* 51.4%) but longer PFS (8.9 *versus* 5.7 months) and received more octreotide LAR treatment (median cycles, 7 *versus* 3). The most common toxicities included grade 3/4 leukopenia/neutropenia (60%), nausea/vomiting (17.5%) and diarrhea (12.5%). Therefore, IP is an active regimen in patients with advanced GEP-PDNEC and should probably not be given to patients with advanced high proliferative NET. The benefit of octreotide LAR maintenance therapy on high proliferation NETs requires further study.

## INTRODUCTION

Gastroenteropancreatic neuroendocrine tumors (GEP-NETs) are a heterogeneous group of neoplasms arising from the diffuse neuroendocrine system. According to the 2010 World Health Organization (WHO) classification [1], neuroendocrine tumors are classified histologically based on tumor differentiation (well or poorly differentiated) and tumor grade (grades 1-3). Poorly differentiated or grade 3 neuroendocrine tumors, which are also called neuroendocrine carcinoma (NEC), have a mitotic count exceeding 20/10 high-power field (HPF) and/or a Ki-67 index exceeding 20%.

Although previously regarded as rare, the incidence of GEP-NEC has recently started to increase [2–6], likely due to more precise pathologic classification. Most GEP-NECs are metastatic at the time of diagnosis, and the patient prognosis is always dismal [7, 8]. Guidelines for the treatment of advanced GEP-NEC advocate the use of platinum-based chemotherapy combined with etoposide [9–12]. However, until recently, only a few small first-line studies have been reported, and the objective response rate (ORR) to the EP regimen for advanced GEP-NECs varies greatly from 14% to 67% [13–15].

In 2002, a clinical trial demonstrated that an irinotecan/cisplatin (IP) regimen achieved better survival than the EP regimen in extensive small cell lung cancer (SCLC) patients [16]. Our retrospective study has also revealed that the ORR of IP chemotherapy was 57.1 %, with a disease control rate of 78.6 % in advanced GEP-NEC patients [17]. Although quite chemo-sensitive, advanced GEP-NECs are always aggressive with short progression-free survival (PFS) and overall survival (OS) times. A procedure that maintains a good response to IP treatment and improves the OS has yet to be determined.

Somatostatin analogs, initially used for the treatment of carcinoid syndrome [18–20], have recently been shown to have anti-proliferative activity [21–23]. In our retrospective study [17], octreotide long-acting release (LAR) was administered as maintenance treatment for patients in which the disease was controlled after an irinotecan/cisplatin regimen. All patients tolerated the octreotide LAR treatment well. The median number of cycles of octreotide LAR was 4.5 (3-20 cycles), which showed that octreotide LAR may be a good option for maintenance treatment.

Hence, we conducted this phase II trial (ClinicalTrials.gov Identifier: NCT01480986) with the aim of investigating the activity and safety of IP treatment and subsequent octreotide LAR treatment as a first-line approach in the treatment of patients with advanced GEP-NECs.

## RESULTS

### Patient characteristics

The study was activated in August 2011 and closed to patient accrual in December 2013. Forty patients were enrolled. Table 1 shows the characteristics of the enrolled patients. In order to guarantee the quality of the trial, all the 5 patients with unknown primary tumor had been reviewed and approved by the multidisciplinary team (MDT) during the screening period. Last follow-up was in December 2014.

**Table 1:** Patient demographics and clinical characteristics

Variables	Number of patients	Proportion (%)
Gender		
Male	30	75.0
Female	10	25.0
Age [median (range)], 56.5 (30–77)		
<59	25	62.5
≥60	15	37.5
Karnofsky performance score(KPS)		
<90	19	47.5
≥90	21	52.5
Location of primary tumor		
Stomach	15	37.5
Esophagus	8	20.0
Pancreas	6	15.0
Colorectum	4	10.0
Small intestine	2	5.0
Unknown primary origin	5	12.5
Histology		
Small cell NEC	20	50.0
Large cell NEC	8	20.0
MANEC	7	17.5
High proliferation NET	5	12.5
Immunohistochemistry(positive/total)		
Synaptophysin	36/40	90.0
Chromogranin A	25/39	64.1
CD56	25/39	64.1
Ki-67		
≥ 75%	21	52.5
50-74%	9	22.5
20-49%	8	20.0
Unknown	2	5.0
Carcinoid syndrome		
Yes	3	7.5
No	37	92.5
Octreotide scanning		
Positive	19	47.5
Negative	20	50.0
Unknown	1	2.5
Stage		
Locally advanced	5	12.5
Metastatic	35	87.5
Metastasis		
Liver	25	62.5
Lung	3	7.5
Bone	5	12.5
Distant lymph nodes	29	72.5
Serum NSE level before treatment		
<50ng/ml	21	52.5
≥50ng/ml	17	42.5
unknown	2	5
Serum LDH level before treatment		
<300U/ml	29	72.5
≥300U/ml	11	27.5

*Abbreviations: NEC=neuroendocrine carcinoma; MANEC=mixed adenoneuroendocrine carcinoma; NET= neuroendocrine tumor; NSE= neuron-specific enolase; LDH= lactic dehydrogenase.

### Treatment received

The total number of IP regimen cycles for all the paitents was 155, with a median of 4 for each patient. Ten patients (25.0%) received only one cycle of IP treatment due to rapid tumor progression (one patient) and treatment-related toxicity (two patients with febrile neutropenia, grade 3 thrombocytopenia and grade 3 diarrhea and seven patients with a treatment delay of more than 2 weeks due to toxicity). Three patients who had locally advanced disease received an R0 resection after systemic treatment. Seventeen patients (42.5%) completed the planned six cycles of IP chemotherapy, and 12 went on to receive octreotide LAR according to the protocol. Four patients received octreotide LAR as maintenance treatment following discontinuing chemotherapy due to severe toxicities. Table 2 shows the characteristics and clinical outcomes of patients with octreotide LAR maintenance treatment. The median number of octreotide LAR cycles was 3 (1-25 cycles). Due to a change in the pharmaceutical dosage forms in China, 6 patients received octreotide LAR (30 mg). Of the 16 patients who received maintenance octreotide LAR, 1 patient received only one dose of octreotide LAR due to intolerable diarrhea, 2 patients discontinued the drug due to financial problems, and the remaining 13 patients continued octreotide LAR treatment until the disease progressed.

**Table 2:** Characteristics and clinical outcomes of patients with advanced GEP-NEC receiving octreotide LAR maintenance treatment

No	Gender /age	Histology	Octreatide scan	Primary site	Metastatic sites	IP cycles	IP response	Octreotide LAR			PFS (m)
								Cycles	Dosage (mg)	Reasons for interruption	
1	F/63	L-NEC	Positive	Pancreas	Liver, LNs	1	NA	3	20	PD	3.27
2	M/55	L-NEC	Positive	colorectum	Cervical LNs	6	PR	3	20	Financial problem	14
3	F/66	S-NEC	Negative	Stomach	Liver, Lung, LNs	6	PR	3	20	PD	5.63
4	M/56	S-NEC	Positive	Unknown	Pelvic and inguinal LNs	4	PR	25	20	PD	26.5
5	M/59	S-NEC	Negative	Unknown	Liver, Bone, LNs	6	PR	3	20	PD	6.9
6	M/51	MANEC	Negative	Esophagus	Lung, Bone, LNs	6	SD	1	20	PD	4.57
7	M/57	MANEC	Positive	Stomach	Liver, LNs	6	PR	2	20	PD	4.53
8	F/48	MANEC	Negative	Stomach	Celiac and retroperitoneal LNs	6	PR	8	20	PD	12.3
9	M/67	MANEC	Negative	Stomach	Liver	6	PR	5	20	PD	7.37
10	M/30	H-NET	Positive	Pancreas	Liver, Bone, LNs	6	SD	5	20	PD	8.93
11	M/54	MANEC	Positive	Stomach	Supraclavicular Celiac, retroperitoneal and pelvic LNs	6	PR	1	30	PD	3.7
12	M/54	L-NEC	Positive	Pancreas	Liver, LNs	6	PR	1	30	Severe diarrhea	5.6
13	F/63	S-NEC	Negative	Unknown	Lung, LNs	3	PR	4	30	PD	5.27
14	M/48	S-NEC	Positive	Pancreas	Liver, LNs	6	SD	4	30	PD	7.6
15	M/56	S-NEC	Positive	Stomach	Liver, LNs	6	PR	3	30	PD	8.17
16	F/55	H-NET	Negative	Stomach	Liver, LNs	4	SD	9	30	Financial problem	14.9

*Abbreviations: F= female; M= male; L-NEC= large cell neuroendocrine carcinoma; S-NEC= small cell neuroendocrine carcinoma; H-NET= high proliferation neuroendocrine tumor; MANEC= mixed adenoneuroendocrine carcinoma; LN= lymph node; IP= irinotecan plus cisplatin; SD= stable disease; PR= partial response; LAR= long-acting release; PD=progression disease; NA= Not applicable; m= month.

After disease progression, the patients were given a variety of palliative treatments including radiotherapy (5 patients, 12.5%), octreotide LAR treatment (2 patients, 5.0%), transcatheter arterial chemoembolization (7 patients, 17.5%) and salvage chemotherapy (9 patients, 22.5%). Salvage chemotherapy regimens consisted of combined chemotherapy including irinotecan with platinum (5 patients, who had got PR or MR in the first line IP treatment), etoposide with platinum, and temozolomide with capecitabine; the single agents included paclitaxel, irinotecan, etoposide and capecitabine.

### Response to treatment

**Figure 1:**
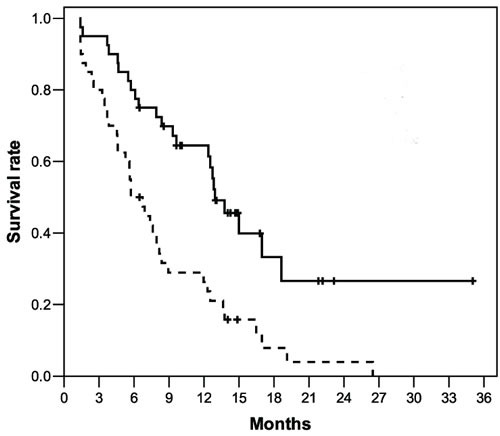
Overall survival (continuous line, median 12.9 months) and progression-free survival (dotted line, median 5.7 months) in the 40 patients with advanced GEP-NEC after IP treatment

A total of 32 patients were evaluable for tumor response. (Table 3) No patient achieved a complete response (CR); eighteen (18/40, 45.0%) patients had a partial response (PR) and 10 (10/40, 25.0%) had stable disease (SD). The disease control rate (DCR) was 70.0%. The response rate to IP treatment in esophageal NEC, gastric NEC, and pancreatic NEC was 62.5%, 40.0% and 16.7%, respectively (*p* = 0.253). The median PFS was 5.7 months (95% confidence interval: 3.8-7.6 months), and the median OS was 12.9 months (95% confidence interval: 10.5-15.4 months) for the entire group. (Figure 1) For the 37 patients who haven't received surgery following IP chemotherapy, the median PFS was 5.6 months (95% confidence interval: 4.3-6.9 months), and the median OS was 12.8 months (95% confidence interval: 10.7-15.0 months).

**Table 3:** Clinical outcomes of IP chemotherapy in patients with advanced GEP-NEC by different histological subtypes

Histology	Response to IP regimen						PFS (months)
	No.of CR (%)	No.of PR (%)	No.of SD (%)	No.of PD (%)	No.of NA (%)	Disease control rate (%)	
Small cell NEC (*n*= 20)	0	11 (55.0)	3 (15.0)	0	6 (30.0)	70.0	6.9
Large cell NEC (*n*= 8)	0	3 (37.5)	2 (25.0)	2 (25.0)	1 (12.5)	62.5	5.6
MANEC (*n*= 7)	0	4 (57.1)	1 (14.3)	1 (14.3)	1 (14.3)	71.4	4.5
High proliferation NET (*n*= 5)	0	0	4 (80.0)	1 (20.0)	0	80.0	8.9
All patients (*n*= 40)	0	18 (45.0)	10 (25.0)	4 (10.0)	8 (20.0)	70.0	5.7

*Abbreviations: IP= irinotecan plus cisplatin; PFS=progression-free survival; CR= complete response; PR= partial response; SD= stable disease; PD= progression disease; NA= Not applicable; NEC= neuroendocrine carcinoma; MANEC= mixed adenoneuroendocrine carcinoma; NET= neuroendocrine tumor.

According to the 2013 Chinese gastrointestinal and pancreatic neuroendocrine neoplasms pathologic consensus [24], compared with the patients in poorly differentiated NEC (PDNEC) group, the patients with high proliferation neuroendocrine tumor (NET) had a lower ORR (0% *versus* 51.4%, *p* = 0.053) but longer PFS (8.9 *versus* 5.7 months) and received more octreotide LAR treatment (median cycles, 7 *versus* 3). (Table 2 and Table 3)

### Toxicities

The IP regimen-related toxicities are summarized in Table 4. Grade 3/4 leukopenia/neutropenia was observed in 24 patients (60.0%), and 3 patients (7.5%) experienced febrile neutropenia. Grade 3/4 thrombocytopenia occurred in 2 patients (5.0%). No grade 3/4 hematological toxicity was observed during the octreotide LAR maintenance treatment.

**Table 4:** Chemotherapy-related toxicity after IP chemotherapy (N = 40; 155 treatment cycles)

Toxicity	All grades Patients, *n* (%)	Grade 3 Patients, *n* (%)	Grade 4 Patients, *n* (%)
Hematologic			
Leukopenia/Neutropenia	35 (87.5)	14 (35.0)	10 (25.0)
Anemia	20 (50.0)	1 (2.5)	0 (0)
Thrombocytopenia	14 (35.0)	2 (5.0)	0 (0)
Neutropenic fever	3 (7.5)	NA	NA
Non-hematologic			
AST/ALT	6 (15.0)	0 (0)	0 (0)
Fatigue	18 (45.0)	1 (2.5)	0 (0)
Neurological sensory	6 (15.0)	0 (0)	0 (0)
Acute cholinergic syndrome	13 (32.5)	0 (0)	0 (0)
Nausea/Vomiting	39 (97.5)	7 (17.5)	0 (0)
Diarrhea	19 (47.5)	5 (12.5)	0 (0)
Anorexia	25 (62.5)	2 (5.0)	0 (0)
Mucositis	3 (7.5)	0 (0)	0 (0)
Alopecia	17 (42.5)	2 (5.0)	0 (0)

*Abbreviations: AST= aspartate aminotransferase; ALT= alanine aminotransferase; NA= Not applicable.

Seven patients (17.5%) suffered from grade 3 nausea/vomiting, 5 patients (12.5%) experienced grade 3 diarrhea, 2 patients (5.0%) exhibited grade 3 anorexia, 2 patients (5.0%) had grade 3 alopecia and one patient (2.5%) had severe fatigue. All other toxicities were mild. Only 1 patient experienced grade 3 diarrhea during octreotide LAR maintenance treatment. Due to the toxicity, nine patients (22.5%) received only one cycle of IP treatment, and fourteen patients (35%) received a dose reduction. No other unexpected toxicities were observed, and no treatment-related death was reported.

## DISCUSSION

Advanced GEP-NEC patients have a poor prognosis. The median survival for patients receiving chemotherapy was 11 months, and for patients without chemotherapy the survival time was only 1 month [7], suggesting that those patients should be considered for chemotherapy without delay.

Many guidelines advocate the use of platinum-based chemotherapy combined with etoposide for the treatment of patients with GEP-NEC, similar to the treatment of small cell lung cancer [9–12]. However, the efficacy of this treatment varies in different studies [13–15]. Mitry et al. [14] retrospectively analyzed the efficacy of the EP regimen in 41 NEC patients; the ORR was 41.5% (4 CR and 13 PR). Grade 3-4 hematological toxicity was observed in 60% of the cases with one treatment-related death. In a recent retrospective study [7], 252 patients with advanced GEP-NEC received cisplatin/etoposide or carboplatin/etoposide; the ORR was only 31%, and the median PFS and OS were 4 and 11 months, respectively. Moreover, in a Japanese retrospective study [15], 21 hepato-biliary-pancreatic NEC patients were treated with EP as a first-line chemotherapy; the ORR, median PFS and OS were only 14% and 1.8 and 5.8 months, respectively, and grade 3 or 4 neutropenia was observed in 90% of these patients.

The JCOG9511 study demonstrated significantly longer OS in the IP arm than in the EP arm for SCLC [16]. In our retrospective analysis of 16 advanced GEP-NEC patients treated with IP, the median PFS and OS were 5.5 and 10.6 months, respectively [17]. Recently, a retrospective Japanese multicenter analysis [25] showed that for patients with advanced NEC of the digestive system including gastrointestinal tract and hepato-biliary-pancreatic system treated with IP/EP (*n* = 160/46), the ORR were 50%/28% and the median OS times were 13.0/7.3 months. A multivariate analysis among patients treated with IP or EP showed a tendency that the efficacy of IP was slightly better than that of EP (HR 0.80, 95% CI 0.48-1.33; *p* = 0.389). In this prospective study, IP as a first-line therapy for advanced stage GEP-NEC resulted in an ORR of 45.0%, a median PFS of 5.7 months and a median OS of 12.9 months, which showed that IP was an active regimen in these patients. Due to the IP treatment-related toxicity, the treatment interruption and dose reduction rates were high. Hence, dose adjustment is necessary in future studies.

To maintain a good response to IP treatment, we searched for an antitumor agent that could be administered for a long period of time with little toxicity. Somatostatin analogs can exert antitumor effects by both direct and indirect mechanisms [26]. A placebo-controlled phase IIIB study (PROMID) [23] demonstrated that octreotide significantly lengthened the time to tumor progression compared to a placebo in patients with treatment-naive metastatic well-differentiated midgut NETs. CLARINET study [27] found Lanreotide was also associated with significantly prolonged progression-free survival among patients with metastatic enteropancreatic neuroendocrine tumors of grade 1 or 2 (Ki-67 < 10%). Till now, there are no data supporting the use of somatostatin analogs in GEP-NEC [6]. Zarogoulidis K et al. found that long acting somatostatin analogues could be used as an additive therapy in combination to antineoplastic agents in patients with SCLC positive for somatostatin receptors [28]. Our retrospective analysis showed that octreotide LAR may be a good option for maintenance treatment [17]. In this prospective study, sixteen patients received octreotide LAR as maintenance treatment. The median cycle (3 cycles) of octreotide LAR treatment was less than that in our retrospective study, and 7 patients received more than three cycles of treatment. Among those patients, one patient with postive Octreatide-Scan received as many as 25 cycles of octreotide LAR, and two patients with negative Octreatide-Scan had 8 and 9 cycles. So, Octreotide LAR did have antitumor effect on the two NEC patients with negative Octreatide-Scan. Indirect antitumor effects of somatostatin analogs, independent of somatostatin receptors, might include inhibition of growth-promoting hormone and growth factor secretion, and antiangiogenic actions [26]. However, due to the insufficient numbers of the population, we can unfortunately not evaluate if there is a benefit of adding octreotide as maintenance therapy after chemotherapy.

According to the 2010 WHO classification, there is an assumption that poorly differentiated histology and high tumor grade are equivalent. However, Scoazec et al. [29], who investigated a series of 104 patients with large cell GEP-NECs, found that 20% of tumors were characterized as well differentiated, despite a Ki-67 index > 20%. In a study of 28 patients with metastatic thoracic and GEP-NECs with a Ki-67 index > 20%, 42.8% of cases had histologically well differentiated tumors. Furthermore, they found that the proportion of patients with short survival ( < 2 years) with G3 well differentiated NETs was smaller than that of patients with G3 large cell NECs (25% *versus* 62.5%, *p* = 0.049) [30]. In addition to the morphology, studies have indicated that a Ki-67 threshold of 55% is predictive for the response to first-line platinum-based chemotherapy. Patients with a Ki-67 index < 55% had a lower ORR (15% *versus* 42%, *p* < 0.001) to platinum-based chemotherapy, but better survival than patients with a Ki-67 index ≥ 55% (14 *versus* 10 months, *p* < 0.001) [7]. These new findings imply that proliferation alone is not sufficient for the classification of high grade GEP-NECs, and the morphology should be incorporated into proliferative indices to correctly classify these neoplasms [31].

Therefore, the 2010 WHO classification may be improved to better define high grade neuroendocrine neoplasms [6, 7, 31, 32]. According to the recommendations of a Chinese pathologic consensus group for gastrointestinal and pancreatic neuroendocrine neoplasms [24], all of the 40 GEP-NEC tumors in this study were re-reviewed by two pathologists independently, and 5 cases were classified as high proliferation NET. This group of tumors shows remarkable differences compared with poorly differentiated GEP-NECs. First, these patients show a different response rate; patients with high proliferation NETs had a much lower ORR to IP (0%) than patients with poorly differentiated NECs (51.4%), which implies that platinum-based chemotherapy may be overused in patients with high proliferation NETs who might benefit from other systemic treatments, such as temozolomide-based or streptozotocin-based chemotherapy, everolimus, sunitinib or others [6, 33]. We recommend that IP treatment should only be given to G3 patients with poorly differentiated morphology. Second, these patients have different octreotide LAR maintenance cycles; patients with high proliferation NETs received more octreotide LAR maintenance cycles than patients with poorly differentiated NECs (median cycles, 7 *versus* 3 cycles). Although the patient numbers in these two subgroups are small, based on the biological behavior, the effect of octreotide LAR maintenance therapy on high proliferation NETs deserve further study. Third, the patients have different prognoses; although they have a lower ORR, patients with high proliferation NETs still had a longer PFS (8.9 *versus* 5.7 months). Therefore, it is very important to analyze the clinical diagnostic and prognostic characteristics and even the molecular pathology and genetic differences between these two subgroups to optimize the treatment of patients with advanced GEP-NECs.

In conclusion, the current WHO high-grade NEC category might needs to be refined. This prospective phase II trial provides additional evidence that IP is an active regimen only in patients with advanced GEP-PDNEC and dose adjustment may be necessary due to toxicity. The observation suggests that IP should probably not be given to high proliferative NET. The benefit of octreotide LAR maintenance therapy on high proliferation NETs requires further study. Future research should focus on these preliminary results in large-scale, randomized trials to assess the ability to improve the treatment response and prognosis of patients with advanced GEP-NECs.

## PATIENTS AND METHODS

### Study design

This was an investigator-initiated, open-label, single-arm phase II clinical study. The primary end point of the study was PFS, which was defined as the time from the date of first treatment until the date of tumor progression or death. The secondary end points included OS, ORR and safety. The OS was defined as the time from the date of first treatment until the date of death. The study was approved by the Medical Ethics Committee of Peking University Cancer Hospital (Beijing, China) and was performed according to the Declaration of Helsinki Principles. Written informed consent was obtained from all patients before inclusion in the study.

### Patients

All patients were enrolled from Peking University Cancer Hospital. Eligible patients had locally inoperable or metastatic, histologically confirmed diagnosis of NEC (Ki-67 index > 20% and/or mitotic count exceeding 20/10 HPF), including mixed adenoneuroendocrine carcinoma (MANEC) if the gland-forming elements exceed 30%, with a gastroenteropancreatic primary or an unknown primary tumor predominantly with gastroenteropancreatic metastases. Additional inclusion criteria consisted of a Karnofsky performance score (KPS) ≥70; at least one radiologically measurable lesion; no previous chemotherapy and adequate bone marrow function; adequate hepatic and renal function; a life expectancy≥90 days; and ≥18 years of age. The exclusion criteria consisted of pregnant or nursing women, uncontrolled severe diarrhea, confirmed or suspected central nervous system metastasis, and a history of myocardial infarction within the past 6 months.

In 2013, a Chinese pathologic consensus group for gastrointestinal and pancreatic neuroendocrine neoplasms proposed a new entity called “high proliferation neuroendocrine tumor (NET)”, which is defined as well differentiated neuroendocrine neoplasms with a Ki-67 level between 20-60% [24]. All of the GEP-NEC tumors in this study were re-reviewed by two pathologists independently for the new classification.

### Pretreatment evaluation

Pre-treatment evaluations included documentation of a routine history, a physical examination, KPS, a complete blood count (CBC), a chemistry profile, measurement of neuron-specific enolase (NSE) and electrocardiography. Tumor staging with computed tomography (CT) scans of the chest and abdomen was required. A magnetic resonance imaging (MRI) scan of the head and octreotide scanning were also required. A bone scan was indicated if the presence of bone metastases was clinically suspected.

### Treatment

All patients received combination chemotherapy with irinotecan (180 mg/m^2^) administered for 90 minutes by intravenous (IV) infusion (day 1) and cisplatin (50 mg/m^2^) administered for 120 minutes by IV infusion (day 1). The treatment courses were repeated every 2 weeks. This regimen required hydration and prophylactic administration of antiemetic drugs. If a patient's neutrophil count was ≤1.0×10^9/L, recombinant human granulocyte colony-stimulating factor (G-CSF) was administered until the neutrophil level was restored.

Responses to treatment were assessed every 6 weeks. Patients who had an objective response or stable disease would receive an additional three cycles of chemotherapy. Patients who had an objective response or stable disease after chemotherapy were treated with octreotide LAR (20 mg, deep intramuscular injection) administered at 4-week intervals. The responses to octreotide LAR maintenance treatment were assessed every twelve weeks. Octreotide LAR was discontinued in cases of unacceptable toxicity, evidence of disease progression or patient refusal.

### Evaluation of the treatment response

Serial tumor assessments, based on the Response Evaluation Criteria in Solid Tumors (RECIST 1.0), were performed every 6 weeks of treatment with a CT scan of the chest and abdomen with or without a scan of the pelvis. After discontinuation of the treatment, routine re-assessment at 6- to 8-week intervals was required for patients who withdrew from the study for reasons other than disease progression.

### Dose modifications

Toxicity was graded using the National Cancer Institution (NCI) Common Toxicity Criteria Version 3.0 (CTC 3.0) by direct questioning, physical examination, measurement of the CBC, and laboratory tests. Chemotherapy was withdrawn until the neutrophil count was ≥1.5×10^9/L, the platelet count was ≥100×10^9/L, diarrhea stopped and other toxicities recovered to ≤grade 1. The dose of irinotecan in subsequent cycles was reduced to 75% of the planned dose if patients experienced grade 4 hematologic toxic effects or if grade 3 diarrhea developed. The dose of cisplatin was reduced to 75% of the planned dose in patients with grade 2 renal toxicity. Treatment was terminated in patients with grade 4 diarrhea, grade 3 or higher renal toxicity, grade 2 or higher pulmonary toxicity, or grade 3 or higher hepatic toxicity.

### Statistical methods

The predicted PFS was the determinant of sample size. On the basis of the varied estimates of the PFS in prior studies [13–15], a PFS of 3.5 months or less was not considered worthy of further investigation. The target enrollment of 32 patients would provide an 80% power to detect a 2-months improvement in PFS with one-sided α of 0.05 and enrollment and follow-up periods of 2.0 and 1.0 years, respectively. To ensure an adequate number of evaluable patients, a total of 40 patients were enrolled.

SPSS (version 13.0) statistical software was used for the statistical analyses. The results are presented as descriptive statistics. The survival curves were generated using the Kaplan-Meier method.
